# The relationship between QT interval indices with cardiac autonomic neuropathy in diabetic patients: a case control study

**DOI:** 10.1186/s13098-020-00609-0

**Published:** 2020-11-19

**Authors:** Maryam Vasheghani, Farzaneh Sarvghadi, Mohammad Reza Beyranvand, Habib Emami

**Affiliations:** 1 Chronic Respiratory Diseases Research Center (CRDRC), National Research Institute of Tuberculosis and Lung Diseases (NRITLD), Shahid Beheshti University of Medical Sciences, Tehran, Iran; 2 Endocrine Research Center, Research Institute for Endocrine Sciences, Shahid Beheshti University of Medical Sciences, Tehran, Iran; 3 Department of Cardiology, Taleghani Hospital, Shahid Beheshti University of Medical Sciences, Tehran, Iran; 4 Tobacco Prevention and Control Research Center, National Research Institute of Tuberculosis and Lung Diseases (NRITLD), Shahid Beheshti University of Medical Sciences, Tehran, Iran; 5 Department of Cardiology, Taleghani Educational Hospital, Tabnak St. Velenjak Region, Chamran High Way, 1985711151 Tehran , Iran

**Keywords:** Cardiac autonomic neuropathy, Diabetes mellitus, Electrocardiogram, ECG, QT interval, QT minimum, QT maximum, QT dispersion, QT mean

## Abstract

**Background:**

Long QT interval (QT) and abnormal QT dispersion (QTd) are associated with sudden death. The relationship between cardiac autonomic neuropathy (CAN) and QT indices in type 2 diabetic patients were investigated.

**Methods:**

Totally 130 diabetic subjects (mean age 50.87 ± 13.9 years) were included (70 individuals with and 60 individuals without CAN). All participants had sinus cardiac rhythm. The patients who had diseases or take drugs that cause orthostatic hypotension (OH), cardiac arrhythmia and QT prolongation were excluded. After interview and examination, standard and continuous ECG was taken in supine position with deep breathing and standing up position. CAN diagnosis was based on Ewing’s tests. QT, QT corrected (QTc), minimum QT (QT min), maximum QT (QT max) and mean ± SD of QT (QT mean) and QTd were assessed from standard ECG. QTc was calculated by Bazett’s formula from V2 lead. QTc > 440 ms in men and QTc > 460 ms in women and QTd > 80 ms were considered abnormal.

**Results:**

In patients with CAN, 21.5% were symptomatic. The prevalence of abnormal QTc and QTd was 11.3% and 28.7%, respectively. There was no significant difference between the patients with or without CAN in terms of long QTc and abnormal QTd. However, the mean ± SD of QT max, QT mean and QTd was higher in the patients with CAN (P value < 0.03). The used cut points for QTc and QTd have high specificity (79% for both) and low sensitivity (30% and 37%, respectively). To use QTc and QTd as screening test for CAN in T2DM patients, the cutoff points 380 and 550 ms are suggested, respectively.

**Conclusion:**

The prevalence of asymptomatic CAN was 3.7 times that of symptomatic CAN. In patients with CAN the QT max, QT mean and QTd were higher than those without CAN. There was no association between CAN and long QTc and abnormal QTd.

## Background

The QT interval (QT) indicates the time needed for ventricular myocardial depolarization and repolarization [1] and several physiological factors influence the duration of QT, such as age and sex, and more importantly, heart rate and autonomic system activity [2]. Therefore QT should be corrected based on heart rate, which is called QTc. The maximum minus the minimum QT interval between the various ECG leads is called QT dispersion (QTd). QTd can be a sign of heterogeneity in the recovery of stimulation phase, and this heterogeneity can be the cause of malignant ventricular arrhythmia [3]. Cardiac autonomic neuropathy (CAN) is prevalent in diabetic (DM) patients and increases the risk of cardiac arrhythmias and events, such as sudden death and myocardial infarction [4]. QTc prolongation has been associated with severity of CAN in DM patients [5]. The main causes of QTc prolongation are long-term diabetes, ischemic heart disease, and autonomic system insufficiency; with less frequency, etiologies such as water and electrolyte imbalance [6]. Long QTc causes serious arrhythmias and sudden death, and, along with nephropathy, increases the mortality rate of patients [7]. Increased QTd is seen in patients who have recent myocardial infarction, long QT syndrome, heart failure and DM with CAN. It can be a cause of malignant ventricular arrhythmias and predict mortality in DM patients [8]. Concerning the effect of CAN on QT, several clinical and experimental studies have shown different effects. Ukpabi OJ showed that QTc is significantly more affected by autoimmune neuropathy than other variables in DM patients [9].

Increased QTd is correlated with CAN. High QTd indicates a dysfunction of the autonomic system of heart in patients with DM [10]. But, QTd have not been associated with CAN even with Holter Monitoring method in some recent studies [11]. In this study the relationship between CAN and QT indices in DM patients was investigated.

## Material and methods

**Fig. 1:**
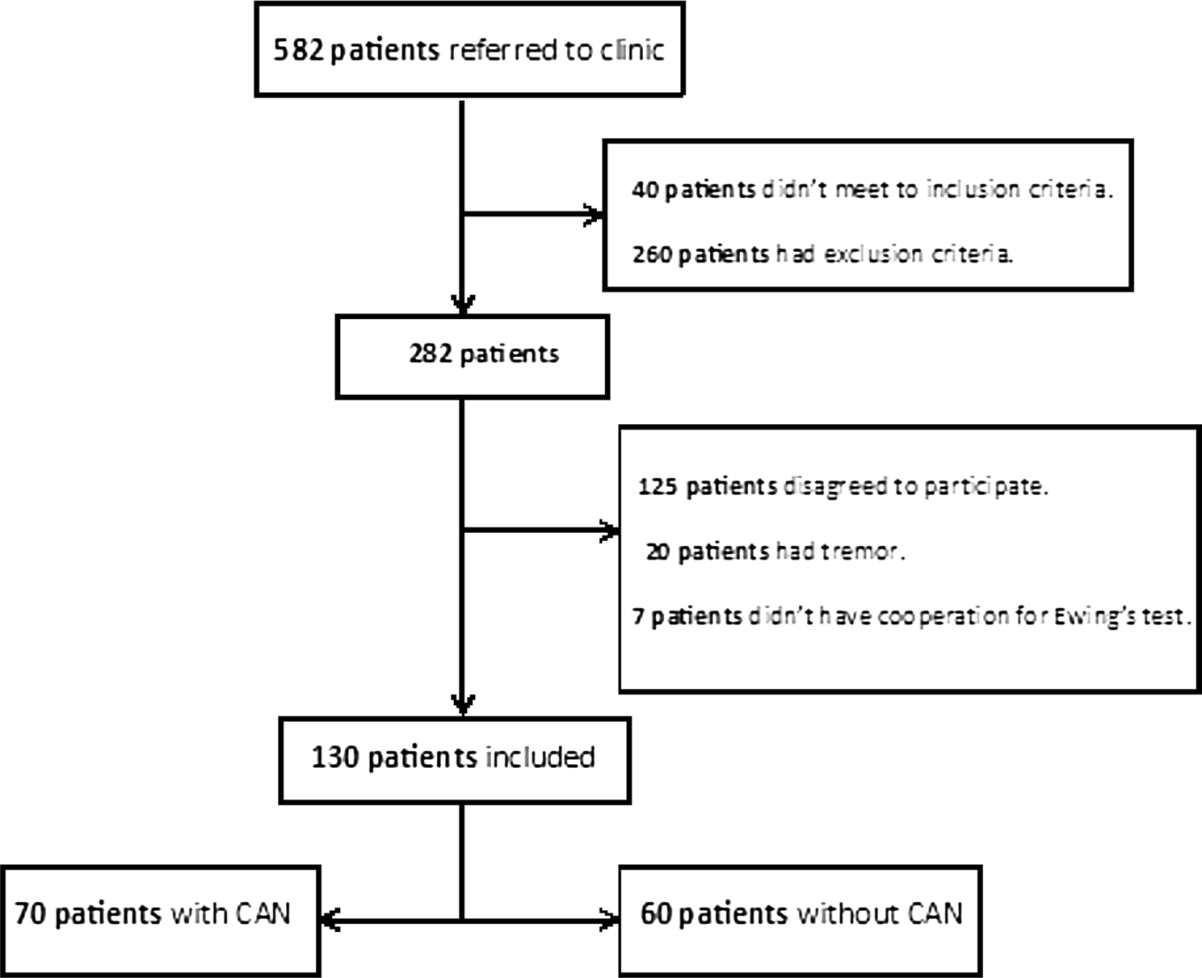
Study design diagram

This cross-sectional study was performed on Type 2 Diabetes Mellitus (T2DM) patients (according to ADA criteria) referred to the internal medicine or endocrine clinic of Loghman Hakim General Hospital in Tehran, Iran [12]. From 582 DM patients who referred during 1 year, 130 patients (70 patients with CAN, 60 patients without CAN) were selected by convenient sampling according to inclusion and exclusion criteria (Fig. 1).

### Inclusion criteria

T2DM patients with cardiac sinus rhythm, normal vital sign and aged 18–75 years old.

### Exclusion criteria

Pregnant women, patients who have symptoms of anemia, hypoxia, hypovolaemia, sepsis, amputation in lower extremity, renal failure, or other diseases affecting the heart rhythm and orthostatic hypotension; taking medications that affect the heart rhythm, QT intervals, and blood pressure. For example: Calcium channel blockers and beta receptors blockers, anti-hypertensive drugs except angiotensin converting enzyme inhibitors and angiotensin receptor blocking agents; anti-arrhythmic drugs and triangular anti-psychotic drugs, Phenotyazine and abnormal difference in blood pressure or arterial pulse between the two arms of the patient.

After receiving written consent, the participants were visited and examined by an internist (Vasheghani M) in fasting state. A questionnaire was completed for demographic information. The patient rests for 15 min and then was examined for blood pressure and heart rate from the right hand in both sitting and standing conditions. Then standard ECG was taken from patients with at least 10 QRS waves per lead. The CAN was assessed based on heart rate variation during physical examination (at rest tachycardia and orthostatic hypotension) and standard Ewing's tests [13]. The details of the research methodology have been published in our previous article [14].

The QT interval demonstrates the distance between the onset of electrical activity and its recovery, which indicates the sympathetic and parasympathetic nervous systems harmony. The QT intervals were calculated based on the standard ECG. The QT interval was calculated from the beginning of the Q wave to the end of the T wave and expressed in milliseconds (ms) [15].

QT minimum (QT min): The minimum QT interval between 12 standard ECG leads.

QT maximum (QT max): The maximum QT interval between 12 standard ECG leads.

QT mean (QT mean): The mean of all QT interval s between 12 standard ECG leads.

$$ {\text{QT Mean}} = \sum {\text{QT intervals}} \, \div {\text{n}};{\text{n}} = {\text{number of QT intervals}} $$

QT corrected (QTc): QTc was calculated based on Bazett's formula from lead V2 of standard ECG. The QTc was considered long if it was more than 460 ms in women and more than 440 ms in men [15].

$$ {\text{Bazett}}^{\prime}{\text{s formula}}:{\text{ QTc}} = {\text{QT interval}} \div {\text{square root of the RR interval }}\left( {\text{in ms}} \right) $$

QT dispersion (QTd): QTd was calculated from the QT max minus QT min. QTd was considered abnormal if it was more than 80 ms [16].

$$ {\text{QTd}} = {\text{QT max}} - {\text{QT min}} $$

This study has been scrutinized at the Ethics Committee of the Faculty of Medicine of Shahid Beheshti University of Medical Sciences and confirmed in accordance with the Helsinki Declaration. The research has been performed according to the opinions of that commission.

### Statistical analysis

The sample size was calculated (53 individual in each group), based on Matel D study data [17]. All data was recorded in the questionnaires. To compare quantitative variables, independent t-test was applied. Chi-square test was applied for qualitative variables. A simple regression analysis was applied to determine the relationship between QT interval indices with each of the quantitative parameters of CAN. Variables normality were tested by Shapiro–Wilk test. Age and BMI had a normal distribution between the two groups and were compared with parametric tests (independent t-test). Duration of DM, fasting blood sugar (FBS), blood sugar 2 h post prandial (BS2hrPP), glycosylated hemoglobin A1C (HbA1C), triglyceride (TG), total cholesterol (TC), high density lipoprotein-cholesterol (HDL-C), low density lipoprotein-cholesterol (LDL-C), 24-h urine protein, QTc, QT min, QT max, QT mean, QT dispersion don't have normal distribution between the two groups and non-parametric tests (Mann–Whitney test) was used. In order to choose best cut-off, we employed Receiver Operating Characteristic (ROC) curve for both QTC and QTd. For statistical analysis, IBM SPSS Statistic version 22 was used and P value less than 0.05 are considered significant.

## Result

Totally, 130 T2DM patients (mean age 50.87 ± 13.9 years) were included (70 individuals with and 60 individuals without CAN). The participant selection is shown in Fig. 1. The prevalence of female sex was 65% vs. 75% in patients with and without CAN, respectively (P-value = 0.9). In patients with CAN, 21.5% were symptomatic and 78.5% were asymptomatic. Patients with CAN had a longer duration of DM, higher BMI, total and LDL-cholesterol level than those without CAN (Table 1). The prevalence of abnormal QTc and QTd was 11.3% and 28.7%, respectively. The Mean ± SD of QT max, QT means and QTd in patients with CAN were higher than those without CAN (Table 2). Area under curve (AUC) for QTc with current cut-off was 0.621 (0.516–0.726, P = 0.028) which was statistically significant. Area under curve (AUC) for QTd with current cut-off was 0.597 (0.490–0.705, P = 0.078) which wasn't statistically significant. Based on the current cut-offs of QTc, it has a sensitivity of 30% and a specificity of 79% in determining the incidence of CAN in DM patients. Based on the current cut-off of QTd, it has a sensitivity of 37% and a specificity of 79% in determining the incidence of CAN in DM patients. In the multivariable regression analysis only total cholesterol had correlation with CAN (OR = 1.014; 95% CI 1.002–1.026, P = 0.02) (Table 3).

**Table 1:** Basic characteristics of diabetic patients with and without CAN

Variables (mean ± SD)	Patients with CAN	Patients without CAN	P_value
Age (years)	49.81 ± 15.52	53.74 ± 11.95	0.35
Duration of DM (month)	*91.67 ± 73.17*	*80.37 ± 79.74*	*0.04*
Fasting blood sugar (mg/dl)	155.12 ± 81.03	178.35 ± 80.12	0.06
Blood sugar 2 h post prandial (mg/dl)	215.14 ± 119.06	233.93 ± 100.67	0.08
HbA1C (%)	7.50 ± 1.72	7.68 ± 1.43	0.21
BMI (kg/m^2^)	*28.06 ± 5.36*	*27.37 ± 4.22*	*0.05*
Total cholesterol (mg/dl)	*220.40 ± 44.36*	*194.72 ± 48.26*	*0.01*
Triglyceride (mg/dl)	173.04 ± 93.31	163.26 ± 141.49	0.06
HDL-C (mg/dl)	51.09 ± 32.87	48.80 ± 17.48	0.45
LDL-C (mg/dl)	*165.52 ± 95.56*	*119.78 ± 40.41*	*< 0.01*
24-h urine protein (mg)	*165.40 ± 472.29*	*88.71 ± 181.58*	*0.70*
Drug history (N)			
Glucose lowering agents			0.54
Oral agent	44	32	
Insulin	20	9	
Diet	2	1	
Lipid lowering agents			0.43
Statins	54	45	
Fibrate	12	16	
Antiplatelete therapy			0.32
Aspirin	46	38	
Clopidogral	9	6	
History of disease (yes, N)			
Current smoker	5	4	0.52
Hypertension	24	14	0.64
Myocardial infarction	5	1	0.39
Stroke	4	2	0.54
Carotid artery stenosis (> 50%)	6	1	0.24
Retinopathy	12	7	0.40
Peripheral neuropathy	34	19	0.80

**Table 2:** The QT intervals indices (mean ± SD) in diabetic patients with and without CAN

Variables (mean ± SD, ms)	Patients with CAN	Patients without CAN	P_value
QT min	340 ± 32	334.7 ± 31	0.80
QT max	*409 ± 34*	*395 ± 34*	*0.03*
QT mean	*375 ± 29*	*365 ± 32*	*0.05*
QTc	415 ± 34	406 ± 32	0.15
QTd	*69 ± 24*	*58 ± 19*	*0.02*

**Table 3:** Multivariate regression analysis of CAN in T2DM patients

Variables	B	Sig	Exp(B)	95% CI for EXP(B)	
				Lower	Upper
HbA1C	− 0.051	0.694	0.950	0.736	1.226
Total cholesterol*	0.014	0.025	1.014	1.002	1.026
Triglyceride	0.003	0.185	1.003	0.999	1.006
LDL-C	− 0.003	0.526	0.997	0.989	1.006
QTc	− 0.074	0.977	0.992	0.006	146.540
QT dispersion	0.014	0.515	1.014	0.995	1.033
QT max	− 0.459	0.501	− 3.271	0.675	1.413
QT mean	0.473	0.511	3.196	0.659	1.048

## Discussion

Long QT intervals and abnormal QTd are associated with sudden death in healthy people, and patients with DM or CAN. So, the relationship between QT indices and CAN in T2DM patients was investigated.

In this study, there was no statistically significant difference between the two groups in terms of age, sex, HbA1C and blood sugar level. Patients with CAN had a longer duration of DM, higher BMI, total and LDL-cholesterol. One-fifth of the subjects had clinical signs (symptomatic CAN). One-third and one-tenth of participants had abnormal QTd and long QTc, respectively. The prevalence of long QTc and abnormal QTd did not differ significantly between two groups. The mean ± SD of the QT max, QT mean and QTd were longer in patients with CAN. There were no significant differences in the mean ± SD of QT min and QTc between the two groups.

Heart rate varies depending on age, sex and circadian cycle [18]. Both groups were similar in terms of age and sex. All measurements were done in the 9:00 AM–4:00 PM and 2 h after waking up. By considering these factors, confounding variables are reduced.

CAN is classified as symptomatic and asymptomatic. The prevalence of symptomatic CAN was 21% in this study, which is consistent with the prevalence of 6% to 32% reported in similar studies [19]. However, the mentioned range is wide, which is due to different methods of blood pressure measuring and criteria for orthostatic hypotension (OH) detection. In various studies, a drop in systolic blood pressure of more than 20 or 30 mm Hg and/or a drop in diastolic blood pressure of more than 10 mm Hg have been used to diagnose OH.

### Long QT and DM

The prevalence of long QTc was 11.3% in this study which is lower than other studies. Like our study, Ninkovic VM studied more than 500 Caucasian patients with T2DM in Serbia [16]. Although all participants of both studies are Caucasian, but he has reported a very high prevalence of long QT (44%). The high prevalence of long QT in their study has several causes. The mean age of participants, male/female ratio, duration of DM, BMI, and percentage of insulin use of their subjects were higher than our study. They also included patients with hypoglycemia. Two separate individuals measured the QT distances with a magnifying glass and equal criteria for long QT is considered for both sexes. Various factors have already been proposed to explain these discrepancy. Long QT may be defined as QT interval > 0.44 ms in either sexes or different value for each sex. Long QT is more prevalent in female, Type 1 Diabetes Mellitus (T1DM), long standing DM and patients with chronic complication of DM [9, 16, 20]. Factors such as race have not yet received especial attention. The prevalence of long-distance QT in blacks, yellows, and Caucasians races was 12% [9], 17% [21], and 44% [16], respectively. However, in a large multicenter study with different nationalities of EURODIAB in 2017, this prevalence was reported to be 17% [22].

### Long QT and CAN

The mean ± SD of QTc did not differ significantly between two groups. This finding is consistent with results of Orosz A and Stern K researches which found that the QT interval in DM and pre-DM individuals was no longer than normal individuals [23, 24]. However, other researchers have found different results. In DM patients with CAN, QTc was significantly higher than those without CAN [25] and prolonged QTc had direct relation to the severity of CAN [26]. The sample size and mean age of their patients were lower and their BMI and glycosylated hemoglobin were higher compare to our study. They had the same criteria for long QT distances in both sexes. The diabetic patients have been compared to healthy people. Long QT intervals may even be seen from the pre-diabetic stage. Lifestyle modification from pre-diabetic stage is more effective in improving the function of the autonomic nervous system [27].

The relationship between QT interval and CAN is very complex. At first, only the association between CAN and the long QTc interval was considered by physicians [28]. It is not clear whether long QT is caused solely by DM or simply due to CAN. Both of them may have a synergistic effect and prolonged QT. The QTc prolongation has been linked to sympathetic and para-sympathetic system activity, and with further research, QT distance was introduced as an index for the diagnosis of CAN and it's severity [29, 30]. There is controversy about this relationship and other studies do not confirm this findings [31]. The long QTc interval may be a risk factor for CAN [32], or considered as a negative consequences of CAN [33]. There are rare reports of short QTc intervals in DM patients with abnormal heart rate variability [34].

It is also theorized that most studies have been performed on people with DM and CAN, and that the long QT distance is more associated with DM than CAN. But, long QT has also been observed in CAN along with other disease such as sickle cell anemia and cirrhosis. This may suggest an independent relationship between long QTc and CAN [35, 36].

Over time, the other measures, such as QTd, QT min, QT max, QT mean and T wave angle, have been used to clarify the relationship between QT interval and CAN. The QT interval variability was introduced as a new diagnostic and/or severity index for CAN. The ratio of QTc variation to heart rate variation determines an index for the balance between QT interval and heart rate variation. This index have been more sensitive for the diagnosis of CAN and the degree of progression of the disease [37, 38].

Although the current used cut-off of QTc has a high specificity and low sensitivity in determining the CAN in DM patients. The criteria used for prolonged QT in this study have two clinical applications, one in the decision of anesthesiologists to induce anesthesia and the other to authorize the initiation of exercise in diabetic patients. Therefore, the degree of specificity is very important for these cases. These cut points are defined for long QT in general population. The QTc in diabetic patients is longer than in healthy individuals in the most studies. Therefore, using a routine cut-offs of QTc does not seem logical. If we want to use them as a CAN screening test, they also have low sensitivity. So, based on the data of this study, a cut-off of 388 ms is suggested for QTC in both sexes with 80% sensitivity and 32% specificity for the screening of CAN in T2DM patients.

### QTd and CAN in DM patients

In this study QTd was 9 ms longer in patients with CAN than those without CAN. This result is in consistent with Statsenko et al. study [39]. Although QTd is longer in patients with T2DM and CAN, but this relation could not be find in the patients with T1DM [40].

Dysfunction of the sympathetic and parasympathetic branches of the autonomic nervous system of the heart increases QTd [41]. The QTd had also a direct correlation with the severity of sensory neuropathy. This difference increased during the standing-up maneuver [39]. Other factors, such as high blood pressure, may affect the results [42, 43].

### QT mean, QT max, QT min and CAN or DM

In this study, the QT min, QT max and QT mean in DM patients with CAN was 6, 5 and 10 ms longer than those without CAN, respectively. Clemente D compared ventricular repolarization in DM patients and healthy individuals. As in our study, the QT max and QT mean in DM patients were higher than healthy individuals (18 and 6 ms, respectively). But, the difference between the two groups was greater than our study [44]. It is important to mention a few points. They compared diabetics with healthy people, but we compared the two groups of diabetics. Participants in their study had older age (mean age = 66 years), better control of DM (mean HbA1C = 7.1%) and both types of DM (11% T1DM).The QT intervals was measured with digital calipers which is more accurate.

Patients with T2DM and CAN also have higher QTc max and QTc mean than those without CAN in Takahashi N [41] and Bankers HR [45] researches as in our study. In patients with CAN, the QT max and QTd were 10 and 40 ms less than in our study, respectively [38]. There are several major differences between the two studies in terms of research methods and participants. The total number of participants and their BMI was lower than our patients. Female to male proportion was equal. Half of the patients injected insulin, while 27% of our patients were treated with insulin. They diagnose CAN with Holter Monitoring and the measurement of HF, LF and LF/HF ratio. The electronic software was used for QT interval measurement. We used Ewing's test for CAN diagnosis and manually QT interval measurements. This method is operator dependent and need to patient collaboration.

Although the current used cut-off of QTc has a high specificity in determining the CAN in DM patients, but its sensitivity is low as a screening test. These routine cut-offs for QTC interval in the general population are not suitable for diagnosing CAN in DM patients. As mentioned before, the QTc distance in diabetic patients is longer than in healthy individuals in the most studies. Therefore, using the routine cut-offs of QTc does not seem logical in this group of patients. Based on the data of this study, a cut-off of 388 ms is suggested for QTC in both sexes with 80% sensitivity and 32% specificity for the screening of CAN in T2DM patients.

The current cut-off of QTd also has low sensitivity in determining the CAN in this study. QTd is longer in patients with diabetes than in healthy people. As mentioned earlier, this finding is more common in type 2 diabetics than in type 1diabetics. In this study, all patients had type 2 diabetes, so it is better to set a specific threshold for this population. According to the results of this study, a cut-off of 550 ms is suggested for QTd with 76% sensitivity and 35% specificity to identify CAN in T2DM patients.

Each of the QT indices may be affected by a part of the nervous autonomic system and one index alone may not show the full function of the autonomic system [41]. We mentioned several items as interfering and confounder factors in this relationship. It is very difficult to clarify the relation between them. To identifying causality relationship, we need to longitudinal cohort study or randomized clinical trials with large sample size and multinational study.

This study has some strength: First, in order to increase the internal validity of the research, all examinations and measurements were performed by one person and with one device. Second, all QT interval related indexes were manually measured. Third, all patients had T2DM. Various factors such as age, gender, and DM control were almost identical between the two groups. Fourth, we suggested a new cut-off for QTc and QTd to identify CAN in T2DM.

The limitations of our study are: First, the study was performed in a single center with a small number of patients. Therefore, the external validity of the study is lower. Second, the sample size was calculated for QTc and may not be sufficient to examine the relationship of other indices to CAN. Third, The QT interval was measured and calculated manually. Therefore, there is a possibility of operator error and the measurements are less accurate than the measurements with computer software. Fourth, other factors which affect QT interval such as electrolyte imbalance (hypo or hyper-kalemia, hypo or hyper-calcemia) were not evaluated.

## Conclusion

This study performed on patients with T2DM in two groups with and without CAN. The asymptomatic CAN was more prevalent than symptomatic CAN. Patients with CAN had a longer duration of diabetes, higher BMI and LDL-cholesterol. The mean ± SD of the QT max, QT mean and QTd were higher in patients with CAN.

## Recommendation

To clarify the relationship between QT distance with diabetes and CAN, it is recommended that multinational and multicenter studies should be conducted. These studies should have a large sample size and be performed in four groups as follows: group I or control, individuals without diabetes and CAN; group II, individuals with diabetes; group III, individuals with CAN; and group IV, individuals with both diabetes and CAN.

To identify CAN in T2DM patients, the new cut-offs of 388 and 550 ms are suggested for QTC and QTd in both sexes.

## Data Availability

The first three authors had access to all the data during the study. Only Dr. Habib Emami, who was added to the group before writing the article and re-analyzed the data, only had access to the data entered in the original SPSS file.
